# Hospital volume and outcomes after code-defined inpatient ventral hernia repair: A machine-learning analysis of the national inpatient sample, 2016–2022

**DOI:** 10.1007/s00423-026-04177-1

**Published:** 2026-08-18

**Authors:** Atef A. Hassan, Ahmed M. Omran, Mohamed Rifai, Mohamed M. Elghor, Abdelnaser Hamdi Mohammed, Tarek Zayid, Sherif Hamdeno, Mohamed Gaafar, Hamzah M. Alghzawi

**Affiliations:** 1 Faculty of Medicine, Al-Azhar University, Cairo, Egypt; 2 Faculty of Medicine, Al-Azhar University, Damietta, Egypt; 3 Faculty of Medicine, Menoufia University, Shibin al Kawm, Egypt; 4 Plastic Surgery Department, Al-Azhar University, Damietta, Egypt; 5 Plastic Surgery Department, Faculty of Medicine, Al-Azhar University, Cairo, Egypt; 6 Plastic Surgery Department, Faculty of Medicine, Al-Azhar University, Assiut, Egypt; 7 School of Nursing, Tennessee State University, Nashville, TN USA; 8 Department of Nursing, Vanderbilt University Medical Center, Nashville, TN USA

**Keywords:** Ventral hernia repair, Hospital volume, Machine learning, Instrumental variables, National inpatient sample, Outcomes research

## Abstract

**Purpose:**

The National Inpatient Sample (NIS) does not capture outpatient hernia surgery or clinical features that define complex incisional hernia. We evaluated the continuous hospital-volume association in an inpatient code-defined abdominal wall repair (CAWR) cohort and assessed its robustness with an instrumental-variable analysis.

**Methods:**

Adults in the 2016–2022 NIS were included when an ICD-10-CM K43.x ventral-hernia diagnosis occurred with an abdominal-wall mesh or component-separation/transversus abdominis release-adjacent procedure code (*n* = 85,426). Double/debiased machine-learning partially linear regression estimated adjusted associations. A leave-one-out Census-division-by-year CAWR-density instrument was used as a sensitivity analysis; its estimate has a local-average-treatment-effect interpretation only if instrumental-variable assumptions hold. The primary outcome combined in-hospital mortality, coded surgical-site infection, and an intensive-care-equivalent severe course; components were also examined separately.

**Results:**

Per 10 additional annual CAWR cases, the adjusted association was − 0.09% points for the composite outcome (95% confidence interval [CI], -0.19 to 0.01; *P* = 0.079) and − 0.05% points for mortality (95% CI, -0.09 to -0.01; *P* = 0.013). Length of stay increased by 0.13 days and charges by approximately 3.1%. In the instrumental-variable analysis, composite and mortality estimates attenuated toward the null, whereas length-of-stay and charge estimates persisted. Component-specific instrumental-variable estimates were heterogeneous.

**Conclusion:**

Within this partial measure of inpatient hospital volume, higher volume was associated with greater inpatient resource use, whereas the mortality association was not robust to instrumental-variable analysis. NIS data cannot establish total institutional hernia volume, anatomical complexity, or a referral threshold.

**Supplementary Information:**

The online version contains supplementary material available at https://doi.org/10.1007/s00423-026-04177-1.

## Introduction

Hospital procedural volume is frequently used as a proxy for surgical quality. Across high-risk operations, higher volume has been associated with lower mortality, but such associations may reflect referral patterns, institutional resources, case selection, and analytic choices rather than an independent volume mechanism [1–8]. Dichotomizing hospitals into high- and low-volume groups also discards the shape of the dose-response relationship and can create arbitrary policy thresholds [9].

Clinically complex incisional hernia is defined by anatomical, wound, procedural-history, and patient factors, including large defects, loss of domain, recurrence, stoma or fistula, active infection, soft-tissue compromise, previous open abdomen or component separation, and selected severe comorbid states [10–15]. These features are not comprehensively available in the NIS. The present cohort is therefore an administrative inpatient phenotype - a K43.x ventral/incisional/parastomal hernia diagnosis accompanied by abdominal-wall mesh or component-separation/TAR-adjacent procedure codes - rather than a validated definition of complex abdominal wall reconstruction [10–12, 16–26]. This distinction is important because mesh reinforcement is routine in many noncomplex repairs and accounted for 95.3% of the cohort.

We modeled annual within-year hospital volume for this code-defined abdominal wall repair (CAWR) cohort as a continuous exposure. Double/debiased machine learning (DoubleML) was used for flexible covariate adjustment [17–19], and instrumental-variable DoubleML was used as an assumptions-based sensitivity analysis rather than proof of causation [20–22, 27]. We hypothesized that a small conventional volume association would attenuate if referral selection, institutional structure, and unmeasured case mix contributed materially to the observed relationship [28–30].

## Materials and methods

### Data source and ethics

We conducted a retrospective cohort study using the HCUP NIS for 2016–2022 [23]. The NIS is a 20% stratified, all-payer sample of discharges from US community hospitals; it does not capture most outpatient or ambulatory-surgery-center repairs. Survey weights, strata, and hospital clusters were used for national descriptive estimates. Because post-2012 HOSP_NIS identifiers are not intended for cross-year linkage, hospital volume was calculated separately within each calendar year. The discharge key was YEAR plus KEY_NIS. Duplicate keys were checked, records missing DISCWT or HOSP_NIS were excluded, and no outcome imputation was performed. The study used de-identified public-use data under the HCUP Data Use Agreement and was exempt from institutional review board review under 45 CFR 46.104(d)(4) [31].

### Cohort

**Fig. 1:**
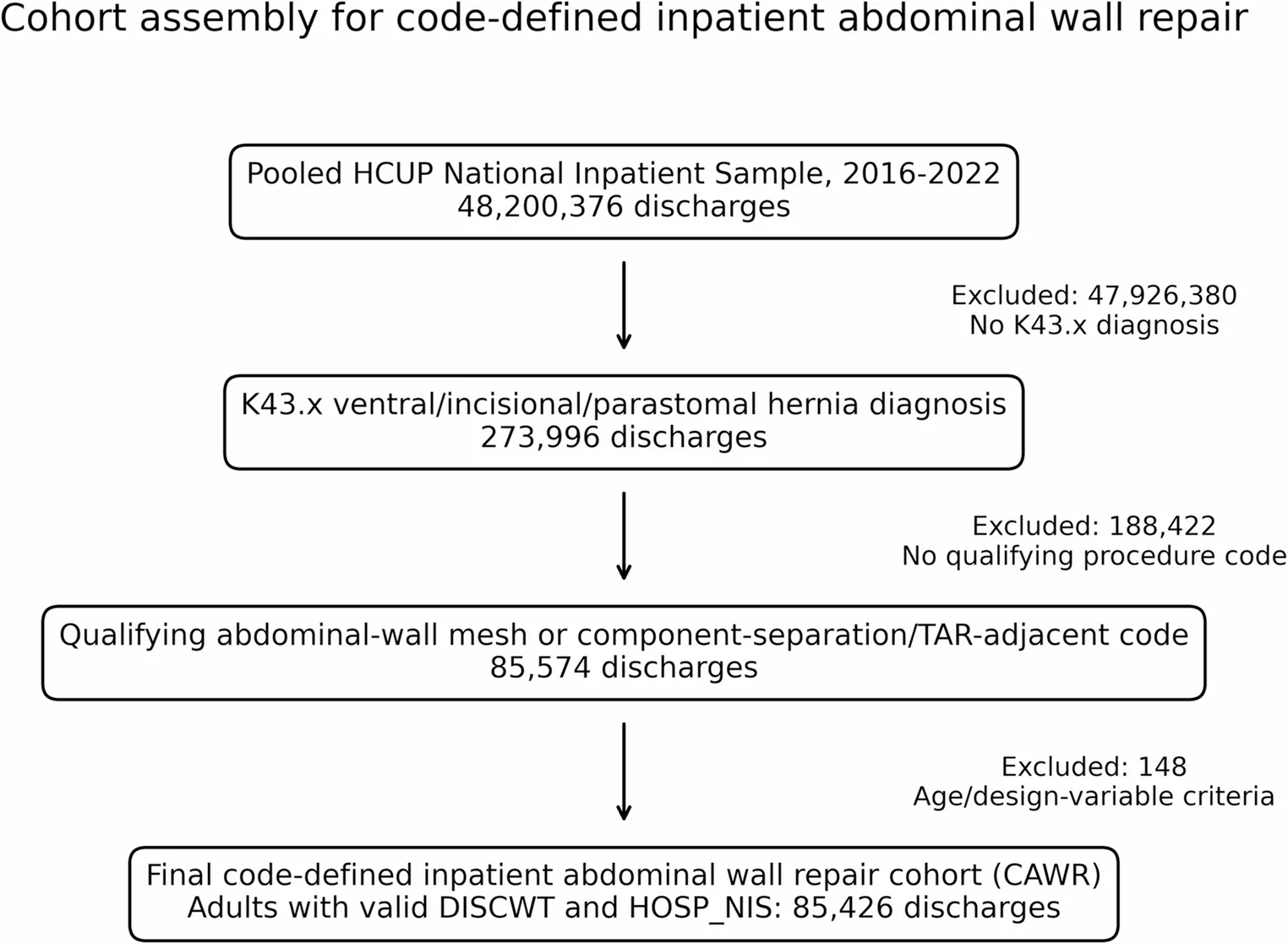
Cohort assembly flow diagram with unweighted counts at each filter step. The pooled 2016–2022 HCUP National Inpatient Sample was filtered by ICD-10-CM K43.x diagnoses and qualifying ICD-10-PCS mesh or component-separation/TAR-adjacent procedures. CAWR, code-defined abdominal wall repair; DISCWT, discharge-level survey weight; HOSP_NIS, within-year hospital identifier

We included adults with an ICD-10-CM K43.0-K43.7 or K43.9 diagnosis plus an ICD-10-PCS 0WUF abdominal-wall mesh supplement code or a component-separation/TAR-adjacent muscle procedure code. “Isolated” inguinal, femoral, umbilical, or diaphragmatic hernia meant a K40, K41, K42, or K44 diagnosis without any K43.x diagnosis; such discharges were excluded to preserve the prespecified K43.x phenotype, not because umbilical hernia is outside the abdominal wall. Full codes and cohort assembly appear in Supplementary Tables S1 and S2 and Fig. 1. Because synthetic-mesh cases dominated the cohort, a higher-complexity proxy sensitivity cohort was restricted to biologic-mesh or component-separation/TAR cases.

### Exposure, instrument, and outcomes

The exposure was the number of qualifying inpatient CAWR discharges at each hospital within each calendar year, modeled per 10 additional cases (median, 3; 95th percentile, 16; maximum, 132; Supplementary Table S3). This exposure does not represent total institutional hernia volume because outpatient, ambulatory-center, nonmesh, and suture-based repairs are absent. The instrument was within-NIS Census-division-by-year leave-one-out CAWR density per 100,000 weighted discharges, excluding the focal hospital contribution. It was selected as a proxy for regional supply and referral opportunity and had a first-stage F statistic of 3955. Its interpretation requires relevance, conditional independence, exclusion restriction, and monotonicity; regional policies, resources, or referral systems could violate the exclusion restriction. The primary outcome was a prespecified severe-course composite comprising in-hospital mortality, coded surgical-site infection (T81.4* or L03.31*), or an intensive-care-equivalent course defined by APR-DRG extreme mortality risk or acute transfer out. Mortality, coded surgical-site infection, the intensive-care proxy, length of stay, and log-transformed total charges were examined separately.

### Covariates and statistical analysis

Prespecified covariates were age, sex, race and ethnicity, payer, ZIP-code income quartile, Elixhauser and Charlson burden, APR-DRG severity, mesh/procedure class, minimally invasive approach, concurrent gastrointestinal resection, elective and weekend admission, hospital bed size, teaching status, hospital ownership/control, Census division, and year. Race and ethnicity were treated as social variables that may capture inequities in access, referral, and hospital selection, not biological attributes; conditioning on them may also attenuate pathways through structural inequality, so subgroup findings were considered exploratory [32, 33]. Bed size and teaching status were retained to estimate the volume association conditional on measured institutional structure. Because these characteristics may also lie on pathways through which volume operates, the adjusted coefficient should not be interpreted as a total causal effect. Risk adjustment followed established administrative-data approaches [34]. The alternative ICD-10 structure-based morbidity and comorbidity scores proposed by Stausberg and Hagn were not used because they were developed in a different coding and validation context; this is considered in the limitations [35, 36].

Survey-weighted characteristics were summarized by hospital-volume quartile. Adjusted associations were estimated with DoubleML partially linear regression, a partialling-out score, XGBoost nuisance learners, and three-fold cross-fitting. DoubleML partially linear instrumental-variable models provided a complementary IV sensitivity estimate. A local average treatment effect interpretation applies only if the instrument assumptions hold; throughout, PLIV results are presented as assumptions-dependent rather than definitive causal estimates. Binary estimates were reported as percentage-point changes per 10 cases; continuous estimates were reported in days or log units. The composite was retained because its individual severe events were uncommon and it summarized adverse index-hospital courses, but component-specific analyses were prespecified because hospital policies and resources may affect the intensive-care proxy differently from mortality or infection. E-values quantified susceptibility of conventional associations to residual confounding [37]. Sensitivity analyses included alternative learners and cross-fitting, empirical-Bayes volume shrinkage, alternative instruments, sampling-weighted M-estimation, elective-only and procedure-specific cohorts, pre-COVID versus COVID-era analyses, placebo outcomes, multiplicity adjustment, and Conley plausibly exogenous bounds. Analyses used Python 3.12, polars 1.38, scikit-learn 1.6, XGBoost 3.0, DoubleML 0.11, and statsmodels 0.14 with a fixed random seed.

## Results

### Cohort and national landscape

The cohort comprised 85,426 unweighted discharges, representing approximately 427,130 national discharges, 4,767 hospitals, and 16,409 hospital-years (Table 1; Fig. 1). Annual qualifying inpatient discharges declined from 14,993 in 2016 to 9,800 in 2022 (Fig. 4; Supplementary Table S4). This trend spans COVID-related surgical disruption [28] but may also reflect migration of ventral-hernia care to outpatient and ambulatory settings; the NIS cannot distinguish these explanations. Synthetic mesh accounted for 95.3% of procedures, whereas biologic mesh and component-separation/TAR accounted for 3.3% and 1.2%, respectively. The crude composite-event rate was 7.37%, mortality was 0.93%, coded surgical-site infection was 1.64%, and the intensive-care proxy was 6.00%. Thus, approximately 77% of composite-positive discharges included the intensive-care proxy, although components could overlap. High-volume hospitals treated more elective cases and were more commonly teaching hospitals (Table 1).

**Table 1:** Baseline characteristics of the code-defined inpatient abdominal wall repair cohort by hospital-volume quartile, NIS 2016–2022

Variable	Overall	Q1 (low)	Q2	Q3	Q4 (high)
N (unweighted)	85,426	4,263	11,353	16,838	52,972
Weighted N	427,130	21,315	56,765	84,190	264,860
Age, mean	60.4	60.7	60.7	60.9	60.1
Female, %	59.6	61.6	61.9	60.7	58.6
White, %	71.8	72.4	69.9	70.1	72.7
Black, %	10.7	9.1	11.0	10.9	10.8
Hispanic, %	10.9	10.4	11.9	12.8	10.1
Medicare, %	46.6	46.4	46.2	47.5	46.4
Medicaid, %	13.6	16.0	15.8	14.5	12.7
Private, %	34.2	31.1	31.5	32.0	35.7
ZIP income Q1, %	27.4	33.1	31.1	27.7	26.1
ZIP income Q4, %	19.8	13.4	16.6	19.1	21.2
Elixhauser sum, mean	2.79	2.68	2.78	2.81	2.80
APRDRG severity, mean	1.96	1.86	1.91	1.95	1.98
Elective admission, %	64.2	49.2	52.7	57.3	70.0
Lap/robotic approach, %	23.2	24.9	26.5	26.6	21.3
Synthetic mesh, %	95.3	97.0	96.4	95.8	94.7
Biologic mesh, %	3.3	2.1	2.5	2.9	3.6
Component-sep/TAR, %	1.2	0.8	1.0	1.2	1.2
Concurrent GI resect, %	17.7	11.5	15.2	16.4	19.1
Teaching hospital, %	75.6	29.2	50.3	65.8	88.0
Length of stay, median	4.0	3.0	4.0	4.0	4.0
Total charges (USD), median	65,777	50,276	59,918	65,183	68,776
Composite event, %	7.37	6.15	7.15	7.64	7.42
In-hospital mortality, %	0.93	0.73	1.13	0.99	0.89
SSI, %	1.64	1.20	1.25	1.50	1.80
ICU proxy, %	6.00	5.11	5.94	6.37	5.96

CAWR, code-defined abdominal wall repair; NIS, National Inpatient Sample; APR-DRG, All Patient Refined Diagnosis Related Group; SSI, surgical-site infection; TAR, transversus abdominis release. CAWR is an administrative inpatient phenotype requiring K43.x plus qualifying mesh or component-separation/TAR-adjacent codes; it is not a validated clinical definition of complex incisional hernia. Hospital-volume quartiles were based on the within-year qualifying inpatient case-count distribution at the hospital-year level (Q1 cut, 1; Q2 cut, 3; Q3 cut, 6 cases/year). Percentages are survey weighted using DISCWT; counts are unweighted

### Volume-outcome estimates

**Fig. 2:**
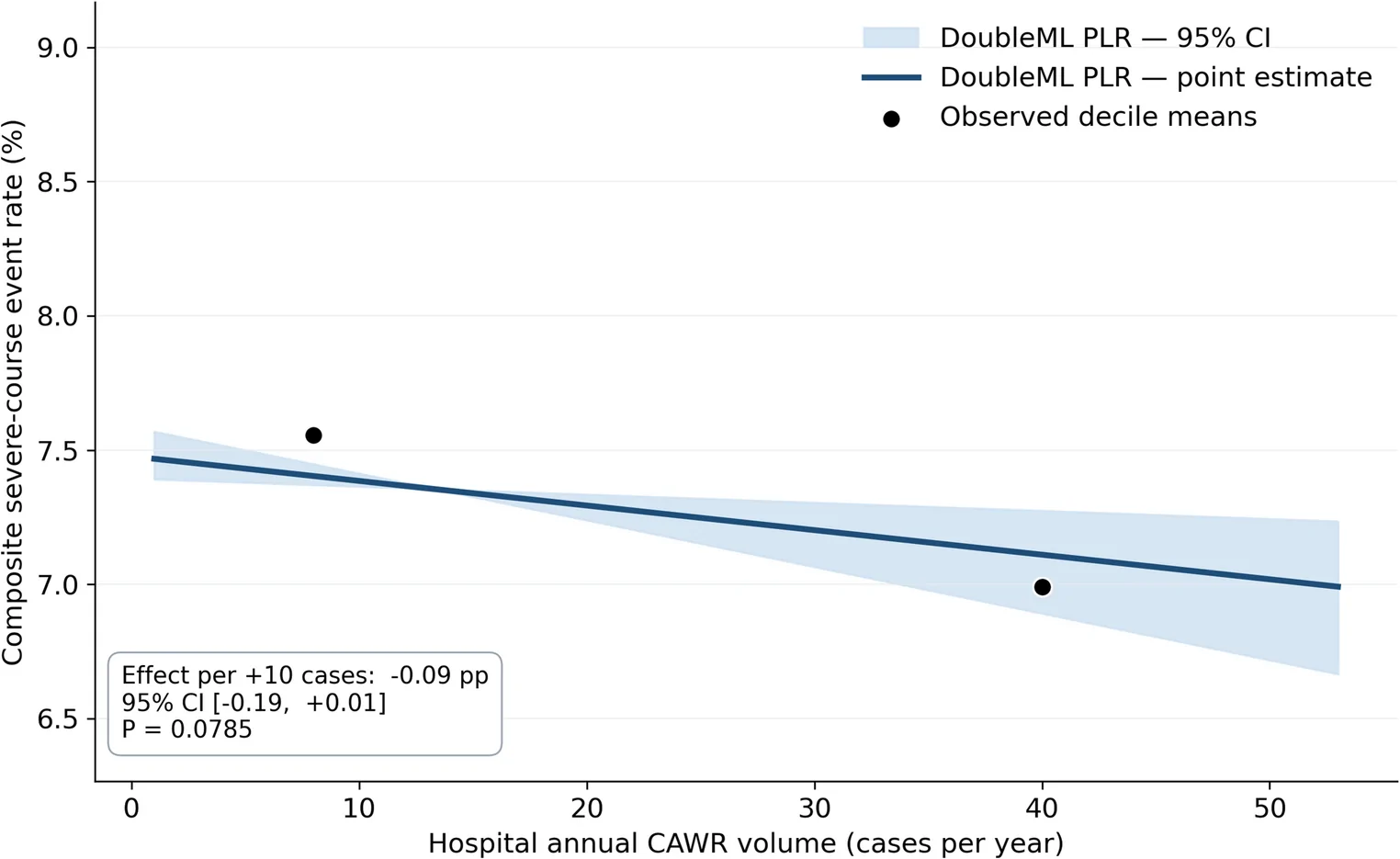
Continuous hospital-volume dose-response curve for the composite severe-course event, estimated by DoubleML partially linear regression with XGBoost cross-fitted nuisance learners and three-fold cross-fitting. The shaded band is the 95% normal-approximation confidence interval; black points are observed survey-weighted volume-group means. The annotation reports the adjusted association per 10 additional annual qualifying inpatient CAWR cases

After cross-fitted adjustment, each 10-case increase in annual qualifying inpatient CAWR volume was associated with a −0.092-percentage-point composite estimate (95% CI, −0.194 to 0.010; *P* = 0.0785) and a −0.053-percentage-point mortality estimate (95% CI, −0.095 to −0.011; *P* = 0.0127) (Fig. 2; Table 2). Length of stay increased by 0.127 days (95% CI, 0.089–0.166), and log charges increased by 0.031 log units, approximately 3.1%. The modeled composite curve was approximately monotonic and did not identify a clinically meaningful threshold.

**Table 2:** Double/debiased machine-learning adjusted associations and instrumental-variable sensitivity estimates of the continuous volume-outcome relationship per 10 additional annual qualifying inpatient CAWR cases

Outcome	Scale	DoubleML PLR (adjusted association)	DoubleML-IV PLIV (IV sensitivity estimate)
Composite severe-course event	percentage points	−0.092 (95% CI, −0.194 to 0.010; *P* = 0.0785)	−0.010 (95% CI, −0.160 to 0.140; *P* = 0.901)
In-hospital mortality	percentage points	−0.053 (95% CI, −0.095 to −0.011; *P* = 0.0127)	−0.020 (95% CI, −0.090 to 0.050; *P* = 0.527)
Length of stay	days	0.127 (95% CI, 0.089 to 0.166; *P* < 0.001)	0.129 (95% CI, 0.073 to 0.184; *P* < 0.001)
Total charges	log units	0.0311 (95% CI, 0.0279 to 0.0342; *P* < 0.001)	0.0310 (95% CI, 0.0265 to 0.0356; *P* < 0.001)

DoubleML used partially linear regression with a partialling-out score, three-fold cross-fitting, and XGBoost nuisance learners (max_depth = 4, n_estimators = 200, learning_rate = 0.05). DoubleML-IV used a partially linear instrumental-variable estimator and within-NIS Census-division-by-year leave-one-out CAWR density per 100,000 weighted discharges (first-stage F = 3955). PLIV estimates are assumptions-dependent sensitivity estimates; a local-average-treatment-effect interpretation requires relevance, conditional independence, exclusion restriction, and monotonicity. Binary estimates are percentage-point changes per 10 cases; continuous estimates are days or log units. CI, confidence interval; PLIV, partially linear instrumental-variable model; PLR, partially linear regression

**Fig. 3:**
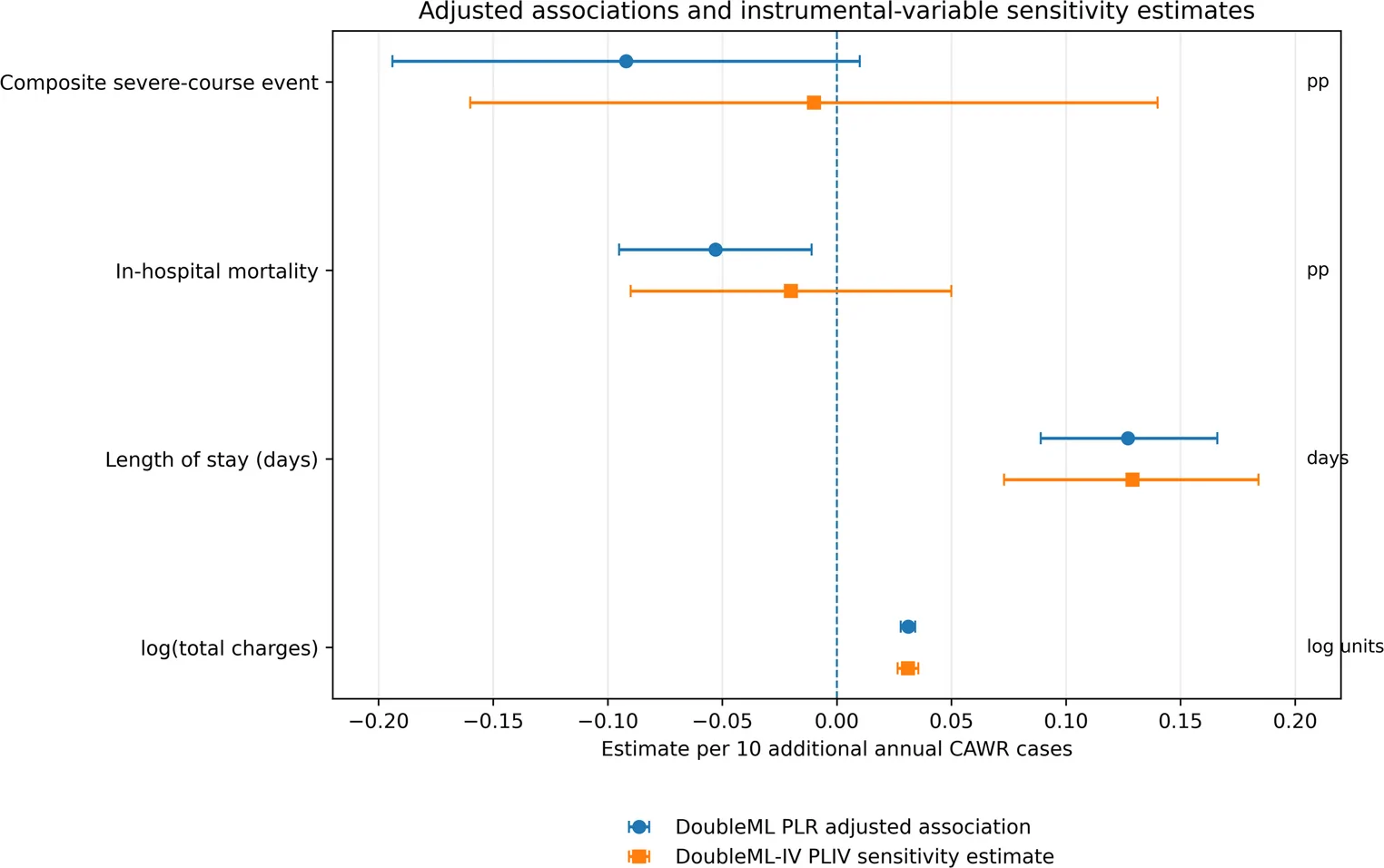
DoubleML adjusted associations and instrumental-variable sensitivity estimates per 10 additional annual qualifying inpatient CAWR cases for four prespecified outcomes. Circles show partially linear regression estimates; squares show partially linear instrumental-variable estimates. The PLIV estimates have a local-average-treatment-effect interpretation only under the stated instrumental-variable assumptions. pp, percentage points

**Fig. 4:**
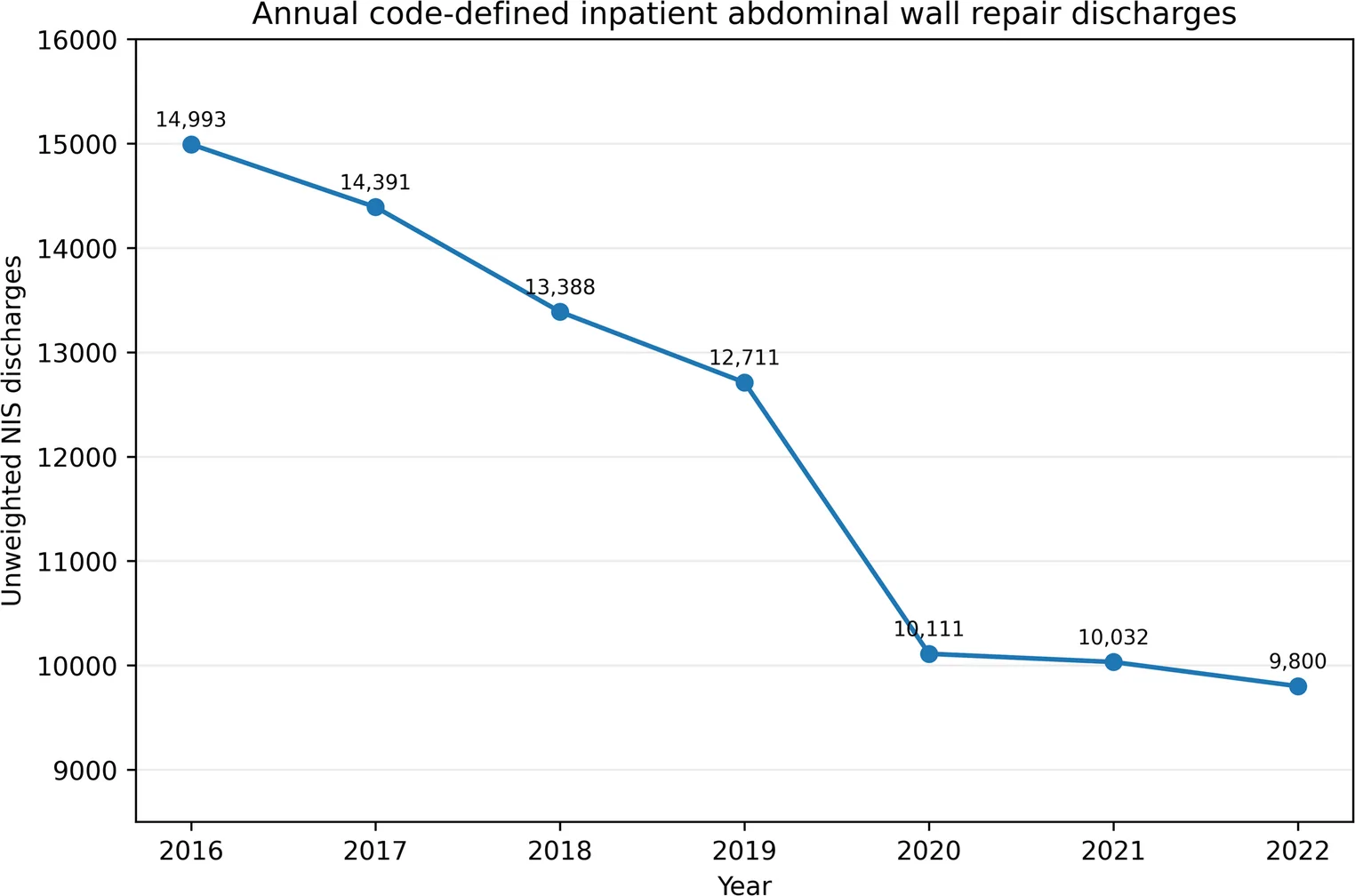
Annual unweighted counts of qualifying code-defined inpatient abdominal wall repair discharges in the NIS, 2016–2022. NIS does not capture most outpatient or ambulatory-surgery-center repairs; the trend therefore cannot be attributed solely to COVID-related disruption or interpreted as total institutional hernia volume

In the instrumental-variable sensitivity analysis, the composite estimate was − 0.010% points (95% CI, −0.160 to 0.140; *P* = 0.901) and the mortality estimate was − 0.020% points (95% CI, −0.090 to 0.050; *P* = 0.527) (Fig. 3; Table 2). Length-of-stay and charge estimates persisted at 0.129 days and 0.031 log units per 10 cases. Component-specific PLIV estimates were heterogeneous: −0.021% points for mortality (*P* = 0.552), + 0.135 for coded surgical-site infection (*P* = 0.0124), and − 0.113 for the intensive-care proxy (*P* = 0.0822) (Supplementary Table S6). These component results reinforce that the composite should not be interpreted as a uniform clinical mechanism. Conventional E-values were modest − 1.12 for the composite and 1.30 for mortality - indicating susceptibility to residual confounding (Table 4; Supplementary Figure S1). (Fig. 4).

### Sensitivity and equity analyses

Length-of-stay and charge associations were stable across prespecified subsets (Supplementary Table S5; Supplementary Figure S3). The adjusted composite association was larger in the higher-complexity proxy cohort (−1.03% points; 95% CI, −1.82 to −0.24), but its PLIV estimate was imprecise and included the null (−0.82% points; 95% CI, −2.00 to 0.36). Five-fold cross-fitting, random-forest learners, and empirical-Bayes shrinkage were close to the primary result; lasso produced a larger linear signal. Alternative instruments were null, and the placebo outcome of patient sex was null (Supplementary Table S6; Supplementary Figure S4). No race/ethnicity or payer association survived Benjamini-Hochberg correction (Table 3; Supplementary Figure S2). (Table 4), [32, 38].

**Table 3:** Equity-stratified estimates of the hospital-volume association with the composite severe-course event, with Benjamini-Hochberg multiplicity adjustment

Subgroup	*N*	Estimate (95% CI)	*P* value	Benjamini–Hochberg q
Race: White	61,283	−0.016 (−0.047 to + 0.015)	0.300	0.741
Race: Black	9,170	−0.025 (−0.110 to + 0.061)	0.572	0.741
Race: Hispanic	9,314	−0.017 (−0.156 to + 0.122)	0.806	0.837
Race: Other	3,378	−0.057 (−0.256 to + 0.142)	0.576	0.741
Payer: Medicare	39,786	−0.015 (−0.051 to + 0.022)	0.430	0.741
Payer: Medicaid	11,631	−0.008 (−0.083 to + 0.067)	0.837	0.837
Payer: Private	29,205	−0.017 (−0.073 to + 0.040)	0.556	0.741
Payer: SelfPay	2,121	+ 0.302 (+ 0.017 to + 0.587)	0.038	0.342
Payer: Other	2,651	−0.087 (−0.342 to + 0.169)	0.504	0.741

Subgroup-specific unpenalized logistic regressions adjusted for the full prespecified covariate set. All subgroups used the full available cohort; subgroups with *N* < 1,500 were excluded. Benjamini-Hochberg q values control the false-discovery rate across all nine displayed strata. Estimates are log-odds coefficients per 10 additional annual qualifying inpatient CAWR cases. Race and ethnicity are social variables; results are exploratory associations, not biological or causal effects

**Table 4:** Residual-confounding E-value bounds for conventional DoubleML partially linear regression associations

Outcome	DoubleML PLR association (95% CI)	E-value (point)	E-value (CI bound)	Interpretation
Composite severe-course event	−0.0009 (−0.0019 to + 0.0001)	1.12	1.00	Residual-confounding RR required to explain the observed association
In-hospital mortality	−0.0005 (−0.0009 to −0.0001)	1.30	1.13	Residual-confounding RR required to explain the observed association

The E-value is the minimum risk-ratio association that an unmeasured confounder would need with both exposure and outcome to explain the observed association, conditional on measured covariates [37]. The CI-bound E-value uses the confidence-limit value closest to the null. CI, confidence interval; PLR, partially linear regression

## Discussion

The central finding is deliberately limited: within a code-defined inpatient ventral-hernia cohort, higher measured hospital volume was associated with longer stays and higher charges, while a small adjusted mortality association was not robust to the assumptions-based IV analysis. These data do not establish that volume independently improves or worsens outcomes, nor do they identify a referral threshold [9].

The exposure is a partial inpatient count rather than total hernia experience. NIS excludes most outpatient and ambulatory-surgery-center repairs and the cohort definition excludes nonmesh or suture-based K43.x repairs. Hospitals that admit a larger share of their hernia patients may therefore appear to have greater volume even when their total operative experience is similar to that of hospitals performing more outpatient care. This limitation can distort both hospital ranking and the estimated volume-outcome association. The annual decline in qualifying NIS discharges consequently cannot be attributed solely to the COVID-19 pandemic or to a true decline in total ventral-hernia repair.

The IV analysis was intended to probe robustness, not to convert an administrative association into causal proof. Regional leave-one-out density is strongly related to focal-hospital volume, but it could affect outcomes through regional resources, transfer practices, access, or other division-level channels. Conley bounds demonstrated limited tolerance for a direct instrument-outcome pathway. Likewise, the composite improved precision for uncommon severe events but was driven predominantly by the intensive-care proxy, which is sensitive to coding, transfer, bed availability, and institutional policy. The heterogeneous component estimates are therefore more informative than a single composite mechanism.

The cohort also cannot be equated with clinically complex incisional hernia. NIS lacks defect width, loss of domain, recurrence history, stoma/fistula, skin or mesh infection, prior open abdomen or component separation, muscle loss, prior flap reconstruction, wound class, mesh plane, BMI thresholds, and cirrhosis with ascites - factors included in the European Hernia Society-endorsed Delphi definition [10–15]. Biomechanically calculated reconstruction, including the GRIP concept, cannot be identified by available ICD-10-PCS codes, and NIS cannot evaluate its reported recurrence- and pain-related durability because follow-up ends at discharge [39, 40]. The higher-complexity proxy analysis therefore remains only a proxy, not a validation of complexity or a comparison of reconstructive techniques.

Additional limitations include unmeasured confounding despite flexible adjustment [41, 42]; possible overadjustment because bed size and teaching status may be mediators; inability to model surgeon volume, cumulative hospital experience, lagged volume effects, or stable cross-year institutional trajectories because HOSP_NIS identifiers are year specific; lack of readmission, recurrence, chronic pain, delayed wound morbidity, patient-reported outcomes, defect morphology, and mesh plane; unvalidated ICD-10 outcome and cohort algorithms; rare mortality; charges rather than costs; and incomplete incorporation of the complex survey design in primary DoubleML models, although weighted sensitivity analyses were performed. Elixhauser and Charlson measures were used, but other ICD-10 structure-based morbidity scores exist and could yield different adjustment [35, 36]. Race/ethnicity adjustment may obscure pathways through structural inequity, and subgroup estimates should not be read as biological or causal effects. Finally, the PLIV exclusion restriction and monotonicity assumptions are untestable, so null IV estimates do not prove absence of a clinically important volume relationship.

In conclusion, higher measured inpatient hospital volume in this code-defined cohort was associated with longer stays and higher charges, whereas the small mortality association was not robust to instrumental-variable analysis. The findings neither support nor oppose referral of selected clinically complex patients to experienced reconstructive teams. They show that NIS-based inpatient volume alone is insufficient to define total institutional experience, causal benefit, or minimum-volume policy. Granular longitudinal registries are needed before referral thresholds or centralization policies can be justified.

## Supplementary Information

Below is the link to the electronic supplementary material.

Supplementary Material 1 (DOCX 1.57 MB)

## Data Availability

No datasets were generated or analysed during the current study.
