# Supplementary material for: Hospital volume and outcomes after code-defined inpatient ventral hernia repair: A machine-learning analysis of the national inpatient sample, 2016–2022
Source: Langenbecks Arch Surg. 2026 Aug 18;411(1):317. doi: 10.1007/s00423-026-04177-1 (PMC13768219; doi:10.1007/s00423-026-04177-1)
Supplement: Supplementary file 1 [file 423_2026_4177_MOESM1_ESM.docx]

**Supplementary Material**

Hospital Volume and Outcomes After Code-Defined Inpatient Ventral Hernia Repair: A Machine-Learning Analysis of the National Inpatient Sample, 2016-2022

Supplementary Tables S1-S12 and Supplementary Figures S1-S11

Scope note. This supplement reports a code-defined inpatient K43.x cohort. NIS does not capture most outpatient or ambulatory-surgery-center repairs and cannot identify the full European Hernia Society complexity criteria, biomechanically calculated reconstruction, surgeon volume, or longitudinal institutional experience. Hospital volume is therefore a within-year count of qualifying inpatient discharges.

# Supplementary Tables

**Table S1. ICD-10-CM diagnosis and ICD-10-PCS procedure codes used for cohort identification. Codes were cross-checked against the 2026 reference set for verification; cohort construction used only billable codes present in the 2016-2022 NIS files.**

**ICD-10-CM diagnosis codes**

| **Code** | **Description** | **Role** |
| --- | --- | --- |
| K43.0 | Incisional hernia with obstruction, without gangrene | Inclusion |
| K43.1 | Incisional hernia with gangrene | Inclusion |
| K43.2 | Incisional hernia without obstruction or gangrene | Inclusion |
| K43.3 | Parastomal hernia with obstruction, without gangrene | Inclusion |
| K43.4 | Parastomal hernia with gangrene | Inclusion |
| K43.5 | Parastomal hernia without obstruction or gangrene | Inclusion |
| K43.6 | Other / unspecified ventral hernia with obstruction, w/o gangrene | Inclusion |
| K43.7 | Other / unspecified ventral hernia with gangrene | Inclusion |
| K43.9 | Ventral hernia without obstruction or gangrene | Inclusion |
| K40 / K41 / K42 / K44 | Inguinal / femoral / umbilical / diaphragmatic hernia | Exclusion (if K43.x absent) |
| T81.4* / L03.31* | Surgical-site / wound infection (ICD-10-CM) | Outcome (SSI flag) |

**ICD-10-PCS procedure codes**

| **PCS code** | **Description** | **Role** |
| --- | --- | --- |
| 0WQF0ZZ | Repair Abdominal Wall, Open | CAWR repair (open) |
| 0WQF4ZZ | Repair Abdominal Wall, Percutaneous Endoscopic | CAWR repair (lap/robotic) |
| 0WUF07Z | Supplement Abdominal Wall — Autologous Tissue, Open | Autologous mesh |
| 0WUF0JZ | Supplement Abdominal Wall — Synthetic, Open | Synthetic mesh — open |
| 0WUF0KZ | Supplement Abdominal Wall — Nonautologous Tissue, Open | Biologic mesh — open |
| 0WUF47Z | Supplement Abdominal Wall — Autologous, Perc Endo | Autologous — lap |
| 0WUF4JZ | Supplement Abdominal Wall — Synthetic, Perc Endo | Synthetic mesh — lap/robotic |
| 0WUF4KZ | Supplement Abdominal Wall — Nonautologous, Perc Endo | Biologic — lap |
| 0KQL0ZZ / 0KQK0ZZ | Repair Left/Right Abdomen Muscle, Open | Component separation |
| 0KML0ZZ / 0KMK0ZZ | Reattachment Left/Right Abdomen Muscle, Open | TAR-adjacent |
| 0JQ80ZZ | Repair Abdomen Subcutaneous Tissue and Fascia, Open | Fascial release adjunct |
| 0DT* / 0DB* | Resection / Excision of GI tract | Concurrent GI resection (contamination proxy) |

Codes are based on the 2026 ICD-10 reference set. The CAWR cohort required a K43.x ventral-hernia diagnosis plus any 0WUF mesh supplement or component-separation/TAR-adjacent muscle code. CAWR denotes a code-defined inpatient abdominal wall repair phenotype and should not be interpreted as a validated clinical definition of complex incisional hernia.

**Table S2. Cohort assembly flow with unweighted counts at each filter step.**

| **Step** | **N (unweighted)** |
| --- | --- |
| NIS 2016-2022 (all discharges) | 48,200,376 |
| Any K43.x ventral-hernia DX | 273,996 |
| + qualifying mesh (0WUF) OR component-separation/TAR-adjacent procedure | 85,574 |
| + adult, valid DISCWT and HOSP_NIS | 85,426 |

Counts are unweighted and discharge-level. The starting NIS denominator was supplied by HCUP. Records missing DISCWT or HOSP_NIS were excluded at the final step. Isolated K40/K41/K42/K44 hernias were those without a concurrent K43.x diagnosis.

**Table S3. Hospital-year CAWR-volume distribution, NIS 2016-2022.**

| **Statistic** | **Value** |
| --- | --- |
| Hospital-year cells | 16,409 |
| Mean (SD) | 5.21 (6.51) |
| Median (IQR) | 3 (1–6) |
| P90 / P95 / P99 | 12 / 16 / 33 |
| Maximum | 132 |

Statistics were computed at the hospital-year level (HOSP_NIS x YEAR). Post-2012 NIS hospital identifiers are not designed for cross-year linkage. Counts include only qualifying inpatient discharges and exclude outpatient, ambulatory-surgery-center, nonmesh, and suture-only repairs.

**Table S4. Annual trends in the code-defined inpatient CAWR procedure mix and crude outcome rates, NIS 2016-2022.**

| **YEAR** | **N (unweighted)** | **N (weighted)** | **Synthetic mesh** | **Biologic mesh** | **Compsep / TAR** | **Lap / robotic** | **Composite** | **Mortality** | **Mean LOS (d)** |
| --- | --- | --- | --- | --- | --- | --- | --- | --- | --- |
| 2016 | 14,993 | 74,965 | 95.64% | 3.28% | 1.05% | 22.92% | 6.27% | 0.89% | 5.72 |
| 2017 | 14,391 | 71,955 | 95.18% | 3.5% | 1.1% | 21.67% | 6.84% | 0.85% | 5.85 |
| 2018 | 13,388 | 66,940 | 95.1% | 3.65% | 1.12% | 23.36% | 7.06% | 0.96% | 5.75 |
| 2019 | 12,711 | 63,555 | 94.98% | 3.37% | 1.2% | 23.72% | 7.66% | 0.87% | 5.67 |
| 2020 | 10,111 | 50,555 | 95.02% | 3.43% | 1.15% | 22.66% | 8.27% | 0.9% | 5.91 |
| 2021 | 10,032 | 50,160 | 95.43% | 2.72% | 1.42% | 23.77% | 8.29% | 0.94% | 6.05 |
| 2022 | 9,800 | 49,000 | 95.49% | 2.67% | 1.33% | 25.02% | 8.01% | 1.2% | 5.89 |

*Counts are unweighted; procedure-mix and outcome percentages are survey weighted.*

**Table S5. Prespecified sensitivity, era, and placebo-outcome analyses across all four outcomes using logistic or linear regression with the full covariate set.**

| **Analysis** | **Outcome** | **N** | **Estimate (95% CI)** | **P value** |
| --- | --- | --- | --- | --- |
| Full cohort (reference) | Composite event | 24,805 | +0.018 (-0.029 to +0.064) | 0.459 |
| Full cohort (reference) | In-hospital mortality | 24,805 | +0.010 (-0.085 to +0.106) | 0.834 |
| Full cohort (reference) | Length of stay (days) | 24,805 | +0.157 (+0.093 to +0.221) | 1.44e-06 |
| Full cohort (reference) | log(total charges) | 24,805 | +0.052 (+0.046 to +0.058) | 1.49e-72 |
| Elective admissions only | Composite event | 24,796 | -0.049 (-0.097 to -0.001) | 0.0477 |
| Elective admissions only | In-hospital mortality | 24,796 | -0.223 (-0.405 to -0.041) | 0.0162 |
| Elective admissions only | Length of stay (days) | 24,796 | +0.097 (+0.051 to +0.143) | 3.3e-05 |
| Elective admissions only | log(total charges) | 24,796 | +0.038 (+0.033 to +0.043) | 1.83e-46 |
| Biologic-mesh only | Composite event | 2,777 | -0.221 (-0.355 to -0.088) | 0.0011 |
| Biologic-mesh only | In-hospital mortality | 2,777 | +0.048 (-0.230 to +0.325) | 0.737 |
| Biologic-mesh only | Length of stay (days) | 2,777 | +0.030 (-0.331 to +0.391) | 0.872 |
| Biologic-mesh only | log(total charges) | 2,777 | +0.066 (+0.048 to +0.084) | 8.12e-13 |
| Component-separation / TAR only | Composite event | 1,000 | +0.104 (-0.054 to +0.263) | 0.198 |
| Component-separation / TAR only | In-hospital mortality | 1,000 | -0.122 (-0.465 to +0.221) | 0.487 |
| Component-separation / TAR only | Length of stay (days) | 1,000 | -0.394 (-1.251 to +0.464) | 0.369 |
| Component-separation / TAR only | log(total charges) | 1,000 | +0.049 (+0.011 to +0.087) | 0.0122 |
| Pre-COVID (2016–2019) | Composite event | 24,817 | -0.039 (-0.091 to +0.012) | 0.136 |
| Pre-COVID (2016–2019) | In-hospital mortality | 24,817 | -0.065 (-0.191 to +0.061) | 0.315 |
| Pre-COVID (2016–2019) | Length of stay (days) | 24,817 | +0.144 (+0.084 to +0.204) | 2.37e-06 |
| Pre-COVID (2016–2019) | log(total charges) | 24,817 | +0.049 (+0.043 to +0.055) | 6.47e-60 |
| COVID era (2020–2022) | Composite event | 24,772 | -0.057 (-0.101 to -0.013) | 0.0116 |
| COVID era (2020–2022) | In-hospital mortality | 24,772 | -0.259 (-0.407 to -0.111) | 0.000615 |
| COVID era (2020–2022) | Length of stay (days) | 24,772 | +0.109 (+0.045 to +0.174) | 0.00092 |
| COVID era (2020–2022) | log(total charges) | 24,772 | +0.042 (+0.037 to +0.047) | 1.04e-56 |
| Placebo: weekend admission | Weekend admission (placebo) | 24,805 | -0.039 (-7.104 to +7.027) | 0.991 |

For binary outcomes, the estimate is the logistic-regression log-odds coefficient; for continuous outcomes, the estimate is the ordinary least-squares coefficient. Large strata were randomly subsampled to N <= 25,000.

**Table S6. Comprehensive robustness panel for the primary composite-event association per 10 additional annual qualifying inpatient CAWR cases.**

| **Analysis** | Estimate (95% CI) | **P value** |
| --- | --- | --- |
| Primary DoubleML PLR (XGBoost, 3-fold) | -0.092 pp (-0.194, +0.010) | 0.0785 |
| DoubleML PLR — 5-fold cross-fitting | -0.092 pp (-0.194, +0.011) | 0.0792 |
| DoubleML PLR — Random Forest learners | -0.100 pp (-0.202, +0.001) | 0.0525 |
| DoubleML PLR — Lasso learners | -0.297 pp (-0.419, -0.175) | 1.77e-06 |
| DoubleML PLR — empirical-Bayes shrunken volume | -0.089 pp (-0.205, +0.027) | 0.134 |
| Higher-complexity proxy cohort (n = 3,746) — PLR | -1.030 pp (-1.817, -0.244) | 0.0103 |
| Higher-complexity proxy cohort — DoubleML-IV (PLIV) | -0.819 pp (-2.001, +0.362) | 0.174 |
| Alt IV — HOSP_REGION × YEAR density (F = 797380) | -0.064 pp (-0.173, +0.044) | 0.246 |
| Alt IV — HOSP_BEDSIZE × YEAR density (F = 502656) | -0.060 pp (-0.170, +0.049) | 0.282 |
| Placebo outcome — patient female sex | +0.000 pp (-0.000, +0.001) | 0.152 |
| Per-component PLIV — in-hospital mortality | -0.021 pp (-0.090, +0.048) | 0.552 |
| Per-component PLIV — coded SSI | +0.135 pp (+0.029, +0.240) | 0.0124 |
| Per-component PLIV — ICU-equivalent severe course | -0.113 pp (-0.241, +0.014) | 0.0822 |
| Conley bounded-IV tipping-point gamma (assumed direct Z-to-Y pathway that shifts the PLIV estimate to zero) | +0.159 pp per +1 SD of Z (+2.2% of baseline) | — |

All probability-scale estimates are percentage points per 10 additional annual CAWR cases. Random-forest learners used 120 estimators, maximum depth 8, and minimum leaf size 10. Lasso used three-fold cross-validated penalty selection. Empirical-Bayes shrinkage weight was 0.88.

**Table S7. TRIPOD+AI applicability assessment for machine-learning nuisance models in an associational study with instrumental-variable sensitivity analysis.**

| **TRIPOD+AI item** | **Applicability to this study** | **Reporting location** |
| --- | --- | --- |
| 1 Title | Partly applicable: title identifies a machine-learning associational analysis; no prediction model was developed. | Title page |
| 2 Abstract | Applicable: objective, data source, estimands, analytic methods, results, and conclusions are summarized. | Abstract |
| 3a Background | Applicable: clinical rationale for flexible nuisance modeling and instrumental-variable identification assumptions is described. | Introduction |
| 3b Prediction objective | Not applicable: the study did not develop or validate a clinical prediction model. | Introduction |
| 4a-4b Data source and dates | Applicable: NIS source, years, and within-year hospital identifiers are reported. | Materials and Methods-Data source and ethics |
| 5a Participants | Applicable: eligibility criteria and clinical setting are specified. | Materials and Methods-Cohort |
| 5b Treatments received | Not applicable as a prediction-model item; procedure classes are reported as cohort features. | Materials and Methods-Cohort |
| 6a Outcome | Applicable in part: associational-analysis outcomes and timing are defined. | Materials and Methods-Exposure, instrument, and outcomes |
| 6b Outcome blinding | Not applicable: outcomes were derived from pre-existing administrative codes. | Materials and Methods |
| 7a Inputs/covariates | Applicable in part: covariates used by nuisance models are listed. | Materials and Methods-Covariates and statistical analysis |
| 7b Predictor blinding | Not applicable: all variables were derived from pre-existing administrative records. | Materials and Methods |
| 8 Sample size | Applicable: all eligible discharges were included; event counts and minimum detectable effects are reported. | Results; Supplementary Material |
| 9 Missing data | Partly applicable: required design-variable exclusions and nonimputation of outcomes are reported. | Materials and Methods-Data source and ethics; Figure 1 |
| 10a-10b Modeling and internal validation | Applicable: algorithms, hyperparameters, cross-fitting, scores, and sensitivity learners are specified. | Materials and Methods; Supplementary Tables S6 and S9 |
| 10c Prediction calculation | Not applicable: no patient-level predictions were produced. | Not applicable |
| 10d Performance measures | Applicable only to nuisance models; out-of-fold discrimination/calibration metrics are reported. | Supplementary Table S9 |
| 11 Risk groups | Not applicable: hospital volume was modeled continuously; quartiles were descriptive only. | Materials and Methods; Table 1 |
| 12 Development versus validation | Not applicable: this was not a prediction-model development/validation study. | Not applicable |
| 13a-13b Participants and characteristics | Applicable: cohort flow, characteristics, and outcome frequencies are reported. | Results; Figure 1; Table 1 |
| 13c Validation comparison | Not applicable. | Not applicable |
| 14 Model-development sample/events | Not applicable as prediction development; analytic sample and outcome rates are reported. | Results |
| 15a Full prediction model | Not applicable: nuisance learners support association and instrumental-variable estimation and are not a deployable model. | Not applicable |
| 15b Model performance | Applicable only to nuisance functions. | Supplementary Table S9 |
| 16 Model updating | Not applicable. | Not applicable |

TRIPOD+AI was developed for clinical prediction models. This study did not develop a prediction model; machine learning was used only for nuisance functions supporting adjusted association and instrumental-variable sensitivity estimates. Prediction-specific items are therefore marked not applicable.

**Table S8. RECORD reporting checklist for studies using routinely collected health data.**

| **Item** | **Checklist item** | **Page / section in manuscript** |
| --- | --- | --- |
| RECORD 1.1 | The type of data used should be specified in the title or abstract | Title / Abstract |
| RECORD 1.2 | Specific type of data source should be reported in title or abstract | Title / Abstract |
| RECORD 1.3 | If applicable, the geographic region and timeframe of database from which records were collected should be reported | Title / Abstract |
| RECORD 6.1 | Detailed description of methods of selection of participants — including filtering based on data quality, completeness, linkage | Methods → Cohort |
| RECORD 6.2 | Validation studies of codes and algorithms used to select participants — citation or original validation | Methods → Cohort |
| RECORD 6.3 | If validation was conducted, results should be reported | Methods → Cohort / Supplementary |
| RECORD 7.1 | Complete list of codes and algorithms used to classify exposures, outcomes, confounders, and effect modifiers should be provided | Supplementary Table S1 |
| RECORD 12.1 | Authors should describe the extent to which the investigators had access to the database population used to create the study population | Methods → Data source |
| RECORD 12.2 | Authors should provide information on the data cleaning methods used in the study | Methods → Statistical analysis |
| RECORD 12.3 | State whether the study included person-level, institutional-level, or other data linkage | Methods → Cohort |
| RECORD 13.1 | Describe in detail the selection of the persons included in the study (i.e., study population selection) including filtering based on data quality, data availability, and linkage | Methods → Cohort / Supplementary Table S2 |
| RECORD 19.1 | Discuss the implications of using data not created or collected to answer the specific research question — including misclassification bias, unmeasured confounding, missing data, and changing eligibility over time | Discussion → Limitations |
| RECORD 22.1 | Authors should provide information on how to access any supplemental information such as the study protocol, raw data, or programming code | Data availability statement |

*RECORD extends STROBE for studies using routinely collected health data.*

**Table S9. Cross-fitted nuisance-model performance across DoubleML folds.**

| **Outcome** | **Nuisance role** | **Learner** | **Fold** | **Metric** | **Metric value** | **Calibration slope** | **Brier score** |
| --- | --- | --- | --- | --- | --- | --- | --- |
| Composite event | Outcome regression (g) | XGBoost | 1 | C-statistic | 0.967 | 1.023 | 0.0309 |
| Composite event | Outcome regression (g) | XGBoost | 2 | C-statistic | 0.966 | 1.025 | 0.0285 |
| Composite event | Outcome regression (g) | XGBoost | 3 | C-statistic | 0.966 | 1.074 | 0.0291 |
| Composite event | Exposure regression (m) | XGBoost | 1 | R² | 0.377 | 1.065 | n/a |
| Composite event | Exposure regression (m) | XGBoost | 2 | R² | 0.377 | 1.026 | n/a |
| Composite event | Exposure regression (m) | XGBoost | 3 | R² | 0.373 | 1.071 | n/a |
| In-hospital mortality | Outcome regression (g) | XGBoost | 1 | C-statistic | 0.955 | 1.013 | 0.0098 |
| In-hospital mortality | Outcome regression (g) | XGBoost | 2 | C-statistic | 0.945 | 0.865 | 0.0073 |
| In-hospital mortality | Outcome regression (g) | XGBoost | 3 | C-statistic | 0.942 | 0.880 | 0.0084 |
| In-hospital mortality | Exposure regression (m) | XGBoost | 1 | R² | 0.377 | 1.065 | n/a |
| In-hospital mortality | Exposure regression (m) | XGBoost | 2 | R² | 0.377 | 1.026 | n/a |
| In-hospital mortality | Exposure regression (m) | XGBoost | 3 | R² | 0.373 | 1.071 | n/a |
| Length of stay (days) | Outcome regression (g) | XGBoost | 1 | R² | 0.358 | 0.971 | n/a |
| Length of stay (days) | Outcome regression (g) | XGBoost | 2 | R² | 0.335 | 0.986 | n/a |
| Length of stay (days) | Outcome regression (g) | XGBoost | 3 | R² | 0.356 | 1.046 | n/a |
| Length of stay (days) | Exposure regression (m) | XGBoost | 1 | R² | 0.377 | 1.065 | n/a |
| Length of stay (days) | Exposure regression (m) | XGBoost | 2 | R² | 0.377 | 1.026 | n/a |
| Length of stay (days) | Exposure regression (m) | XGBoost | 3 | R² | 0.373 | 1.071 | n/a |
| log(total charges) | Outcome regression (g) | XGBoost | 1 | R² | 0.442 | 1.042 | n/a |
| log(total charges) | Outcome regression (g) | XGBoost | 2 | R² | 0.428 | 1.014 | n/a |
| log(total charges) | Outcome regression (g) | XGBoost | 3 | R² | 0.445 | 1.066 | n/a |
| log(total charges) | Exposure regression (m) | XGBoost | 1 | R² | 0.377 | 1.065 | n/a |
| log(total charges) | Exposure regression (m) | XGBoost | 2 | R² | 0.377 | 1.026 | n/a |
| log(total charges) | Exposure regression (m) | XGBoost | 3 | R² | 0.373 | 1.071 | n/a |

*XGBoost used max_depth = 4, n_estimators = 200, learning_rate = 0.05, and the histogram tree method; three-fold cross-fitting and random seed 42 were used. Binary outcomes report C statistic, calibration slope, and Brier score; continuous outcomes report R-squared and calibration slope.*

**Table S10. Sensitivity of primary estimates to NIS sampling-weighted M-estimation.**

| **Outcome** | **Estimator** | **Estimate per +10 cases (95% CI)** | **P value** |
| --- | --- | --- | --- |
| Composite event | DoubleML PLR (unweighted, primary) | -0.092 pp (-0.194, +0.010) | 0.0785 |
| Composite event | Sampling-weighted LPM (DISCWT, HC1 robust) | -0.292 pp (-0.414, -0.170) | 2.79e-06 |
| In-hospital mortality | DoubleML PLR (unweighted, primary) | -0.053 pp (-0.095, -0.011) | 0.0127 |
| In-hospital mortality | Sampling-weighted LPM (DISCWT, HC1 robust) | -0.104 pp (-0.144, -0.064) | 3.61e-07 |
| Length of stay (days) | DoubleML PLR (unweighted, primary) | +0.127 days (+0.089, +0.166) | 9.32e-11 |
| Length of stay (days) | Sampling-weighted WLS (DISCWT, HC1 robust) | +0.092 days (+0.054, +0.130) | 1.91e-06 |
| log(total charges) | DoubleML PLR (unweighted, primary) | +0.031 log-units (+0.028, +0.034) | 2.42e-81 |
| log(total charges) | Sampling-weighted WLS (DISCWT, HC1 robust) | +0.029 log-units (+0.026, +0.032) | 1.3e-74 |

*Weighted models used DISCWT frequency weights and HC1 heteroscedasticity-robust standard errors. Binary outcomes used weighted linear probability models; continuous outcomes used weighted ordinary least squares.*

**Table S11. Family-wise multiplicity sensitivity across the four primary and secondary outcomes.**

| **Outcome** | **Raw P value** | **Bonferroni-adjusted (k=4)** | **Holm-adjusted** | **Benjamini–Hochberg q** |
| --- | --- | --- | --- | --- |
| Composite event | 0.079 | 0.314 | 0.079 | 0.079 |
| In-hospital mortality | 0.013 | 0.052 | 0.026 | 0.017 |
| Length of stay (days) | <0.001 | <0.001 | <0.001 | <0.001 |
| log(total charges) | <0.001 | <0.001 | <0.001 | <0.001 |

*Bonferroni, Holm, and Benjamini-Hochberg adjustments were applied across four outcomes.*

**Table S12. Conley plausibly exogenous bounded-IV sensitivity across gamma values for all four outcomes.**

| **Outcome** | **γ in SD(Z) units** | **PLIV estimate (per +10 cases)** |
| --- | --- | --- |
| Composite event | -2.0 | -2.0311 pp |
| Composite event | -1.5 | -1.5502 pp |
| Composite event | -1.0 | -1.0692 pp |
| Composite event | -0.5 | -0.5882 pp |
| Composite event | +0.0 | -0.1073 pp |
| Composite event | +0.5 | +0.3737 pp |
| Composite event | +1.0 | +0.8546 pp |
| Composite event | +1.5 | +1.3356 pp |
| Composite event | +2.0 | +1.8166 pp |
| In-hospital mortality | -2.0 | -1.9423 pp |
| In-hospital mortality | -1.5 | -1.4612 pp |
| In-hospital mortality | -1.0 | -0.9800 pp |
| In-hospital mortality | -0.5 | -0.4988 pp |
| In-hospital mortality | +0.0 | -0.0177 pp |
| In-hospital mortality | +0.5 | +0.4635 pp |
| In-hospital mortality | +1.0 | +0.9446 pp |
| In-hospital mortality | +1.5 | +1.4258 pp |
| In-hospital mortality | +2.0 | +1.9069 pp |
| Length of stay (days) | -2.0 | +0.0185 days |
| Length of stay (days) | -1.5 | +0.0233 days |
| Length of stay (days) | -1.0 | +0.0281 days |
| Length of stay (days) | -0.5 | +0.0329 days |
| Length of stay (days) | +0.0 | +0.0377 days |
| Length of stay (days) | +0.5 | +0.0425 days |
| Length of stay (days) | +1.0 | +0.0473 days |
| Length of stay (days) | +1.5 | +0.0521 days |
| Length of stay (days) | +2.0 | +0.0570 days |
| log(total charges) | -2.0 | -0.0178 log-units |
| log(total charges) | -1.5 | -0.0130 log-units |
| log(total charges) | -1.0 | -0.0082 log-units |
| log(total charges) | -0.5 | -0.0034 log-units |
| log(total charges) | +0.0 | +0.0013 log-units |
| log(total charges) | +0.5 | +0.0061 log-units |
| log(total charges) | +1.0 | +0.0109 log-units |
| log(total charges) | +1.5 | +0.0157 log-units |
| log(total charges) | +2.0 | +0.0204 log-units |

*Gamma is expressed in standard-deviation units of the residualized standardized instrument. Beta is the corresponding IV estimate per 10 additional annual CAWR cases.*

# Supplementary Figures

Figure S1. Residual-confounding bounds (E-values) for the conventional DoubleML PLR composite-event and in-hospital-mortality associations. The dashed line marks an E-value of 2 as a modest reference threshold.

Figure S2. Equity analysis. Subgroup-stratified composite-event estimates per 10 additional annual CAWR cases from adjusted logistic regression, shown by race/ethnicity and primary payer. Estimates are exploratory and should be interpreted with multiplicity-adjusted Table 3.

**Figure S3. Sensitivity-analysis forest plot. Per-10-case adjusted logistic-regression coefficients for the composite event and in-hospital mortality across prespecified cohorts and eras. The placebo weekend-admission analysis is shown at bottom.**

Figure S4. Robustness panel for the composite-event estimate. Circles show PLR specifications, squares show instrumental-variable PLIV sensitivity specifications, and the diamond shows the placebo outcome. PLIV estimates are assumptions-dependent and are not presented as a definitive causal interpretation.

**Figure S5. Annual trends in procedure mix and minimally invasive approach during 2016-2022. Synthetic mesh accounted for approximately 95% of qualifying inpatient cases, biologic mesh for approximately 3%, and component separation/TAR for approximately 1%. These categories do not establish clinical hernia complexity.**

**Figure S6. Hospital-year CAWR-volume distribution on a log1p scale. Quartile cut points and the median are annotated; the distribution was strongly right-skewed.**

**Figure S7. Crude survey-weighted composite-event and in-hospital-mortality rates by hospital annual CAWR-volume decile, plotted on a log-scale x-axis.**

**Figure S8. Survey-weighted composite-event rate by US Census division and hospital-volume quartile.**

**Figure S9. DoubleML continuous dose-response curve for in-hospital mortality. The shaded band is the 95% confidence interval; black points are observed survey-weighted volume-group means.**

**Figure S10. DoubleML continuous dose-response curve for length of stay. The shaded band is the 95% confidence interval; black points are observed survey-weighted volume-group means.**

**Figure S11. DoubleML continuous dose-response curve for log(total charges). The shaded band is the 95% confidence interval; black points are observed survey-weighted volume-group means.**
